# Smuts to the Power of Three: Biotechnology, Biotrophy, and Basic Biology

**DOI:** 10.3390/jof7080660

**Published:** 2021-08-14

**Authors:** Jan Schirawski, Michael H. Perlin, Barry J. Saville

**Affiliations:** 1 Matthias-Schleiden-Institute/Genetics, Friedrich-Schiller-University Jena, 07743 Jena, Germany; 2 Department of Biology, Program on Disease Resistance, University of Louisville, Louisville, KY 40292, USA; 3 Forensic Science, Master of Science Forensic Science, and Environmental and Life Sciences Graduate Programs, Trent University, Peterborough, ON K9L 0G2, Canada

## 1. Introduction

Smut fungi are a large group of mainly biotrophic plant pathogens, many of which cause disease on cereal crops. They are amenable to genetic analysis and molecular manipulation, which enables their use as models to study basic biological questions as well as strategies of plant infection. In addition, they serve as vehicles to investigate plant or fungal protein function—also of species that are less tractable and have recently been developed to serve as biotechnological agents for the production of chemicals and proteins. Beginning with the maize smut fungus, *Ustilago maydis*, research interests have broadened to include a diversity of species. This Special Issue on “Smut Fungi” presents 19 articles, in which at least eight different smut species have been used for investigations that reflect up-to-date research in the fields of biotechnology, biotrophy, and basic biology. Each of these topics holds surprises, so: be excited, be curious, and be inspired.

## 2. Biotechnology—Smuts for Technology and Technology for Smuts

Using fungi for the biologic production of chemicals and proteins is a well-established practice in biotechnology. The use of smut fungi for this purpose is in its infancy. In this issue, you will find three articles that uncover the potential of using *U. maydis* to produce chemicals and proteins. L. Ullmann and coworkers screened 72 different strains of the Ustilaginaceae for the ability to co-consume the CO_2_-derived chemicals acetate and formate for the generation of itaconate, a dicarboxylic acid used for the production of biopolymers. They identified one *U. maydis* (MB215) and one *Ustilago rabenhorstiana* strain that were able to use acetate and glucose as co-substrates to produce itaconate, and one *Ustilago cynodontis* strain that utilized formate as a co-substrate for itaconate generation. This research paves the way for realizing the dream of carbon-neutral itaconate production [1]. J. Becker and coworkers went a step further and genetically optimized the metabolism of the identified *U. maydis* MB215 strain for itaconate production by combining several genetic manipulations in a single strain. This led to itaconate production at maximal theoretical yield carried out under biotechnologically relevant culturing conditions [2]. A different application was established by K.P. Hussnaetter and coworkers, who developed *U. maydis* into a regulatable protein production system in which heterologous protein biosynthesis can be uncoupled from protein export and release. They used unconventional secretion of the chitin synthase Cts1 that was expressed only after biomass generation coupled to regulated Don3-mediated product release for production of GFP nanobodies, thereby showing that *U. maydis* can be used for heterologous production of high-value proteins [3].

Three additional articles provided methods for the manipulation of smuts. S.-M. Wege and coworkers show the successful application of CRISPR/Cas9 technology for targeted gene deletion, introduction of point mutations, heterologous complementation at the native genomic locus, and endogenous N-terminal protein tagging in *U. maydis* that only require transient expression of the Cas9hf endonuclease and the designed sgRNA [4]. Application of CRISPR/Cas9 is also possible for *Ustilago hordei*, a smut fungus forming intracellular structures with high similarity to haustoria of obligate rust fungi. B. Ökmen and coworkers developed a solopathogenic strain and suggest its use for the functional study of heterologous rust effector proteins [5]. In contrast, L. Plücker and coworkers suggest development of a model of the Brassicaceae smut fungus *Thecaphora thlaspeos* that can infect *Arabidopsis thaliana*. They established a fungal transformation protocol and successfully generated a gene deletion strain, thereby providing a smut–plant pathosystem where both interaction partners are amenable to genetic analysis and molecular manipulation [6].

## 3. Biotrophy—Smuts Entering and Living in the Plant Realm

Biotrophic fungi spend all or some of their life in close association with living plant tissue, often living within plant cells. This environment imposes specific demands on the fungus, since it needs to dampen the plant immune responses, to recognize specific plant tissues, and to have some way of finding the correct tissue for multiplication and fungal teliospore development. In this issue, four research articles and four reviews address questions regarding how this intimate relationship is established, maintained, and altered during fungal and plant development. B. Zhang and coworkers describe the fungal gene expression response of *Sporisorium reilianum* to colonization of resistant and susceptible maize near-isogenic lines that differ solely in the presence of the resistance gene ZmWAK. They found that expression of ZmWAK successfully blocked the expression of fungal genes coding for secreted proteins [7]. Dual transcriptome data of resistant and susceptible sugarcane plants infected with *Sporisorium scitamineum* were used by N.S. Teixeira-Silva and coworkers to identify and investigate selected candidate effector proteins to determine their stage- and tissue-specific gene expression, subcellular localization, and possible plant interaction partners [8]. A specific fungal effector (UhAVR1) of *Ustilago hordei* that functions as an avirulence protein in barley plants carrying the resistance gene *RUH1* was the object of study by A.P. Montenegro Alonso and coworkers. They found that the effector protein is required for virulence on susceptible barley, induced a hypersensitive reaction (HR) in resistant barley cultivars, was secreted via the ER–Golgi pathway, located to the cytosol when expressed in plant cells, and had a role in suppressing conserved plant basal immunity components [9]. Effectors of *U. maydis* were the object of study of J.R.L. Depotter and coworkers. They compared effector gene expression and effector gene conservation to *S. reilianum* and found that highly conserved effectors were expressed at pre-/penetration stages, while more divergently evolving effectors were expressed during plant colonization. The same separation into conservatively and divergently evolving effectors was found when sampling a collection of wild *U. maydis* isolates, confirming that effectors evolve under different selection pressures [10]. 

In addition to the four research articles, four reviews highlighting different aspects of the biotrophic smut–plant interaction enrich this issue. C. Vicente and coworkers summarize the pre-infection events needed for successful colonization of *S. scitamineum* on sugarcane. For this fungus, a mechanism of spore aggregation, putatively involving quorum sensing as well as cytoskeletal components, is suggested to be an important prerequisite for plant infection [11]. M.D. Pejenaute-Ochoa and coworkers focus on protein O-mannosyltransferases (Pmts) and their role during fungal plant pathogenicity. Including *U. maydis* Pmt4 that is essential for virulence, the authors draw a comprehensive picture of structural, evolutionary, and functional aspects of the Pmt family of proteins in fungi and beyond, which highlights the enormous relevance of these glycotransferases for fungal pathogenic development [12]. In their review, K. van der Linde und V. Göhre also take a look at the larger picture, but in a very different dimension. They summarize models and ideas on how the fungal hyphae may orient themselves in time and space once inside the plant, thereby pointing out important knowledge gaps that need to be closed through further research [13]. Further research avenues and open questions on organ-specific maize infection by *U. maydis*, and how to tackle them using mutants of both the plant and the fungus, are presented in the review by A.C. Ferris and V. Walbot [14]. Since the methods and tools are available, the remaining question is: who is going to do the experiments?

## 4. Basic Biology—Smut Basic Biology Understood

In this issue, five articles address very basic biological questions. J. Petkovic and coworkers observed that in contrast to *Saccharomyces cerevisiae*, *U. maydis* stationary phase cells left in water suffer a dramatic loss of viability that can be restored by aeration. They found that loss of viability was due to an oxygen-consuming cellular activity that led to the killing of most cells due to oxidative stress. The few survivors were able to repopulate upon aeration and were also able to resume growth under hypoxic conditions, suggesting that the remnant cells switched to a fermentative mode of growth [15]. D. Matuz-Mares and coworkers investigated the formation of mitochondrial respiratory supercomplexes of *U. maydis*. Supercomplex formation of respiratory chain complexes is thought to help in cellular adaptation to conditions of increased energy need. They found the existence of four distinct supercomplexes (associations of complex I_1_ with III_2_, I_1_ with IV_1_, I_1_ with III_2_ and IV_1_, as well as I_1_ with III_2_ and IV_2_) that were not found to associate with complex II, the alternative NADH dehydrogenases, or with ATP synthase, and also were not affected by growth conditions [16]. Zooming out to the population level, M.S. Gurjar and coworkers investigated the similarity of *Tilletia indica* isolates of a recent wheat karnal bunt outbreak in India. Using multilocus sequence typing and single nucleotide polymorphism analysis, they showed that the investigated population was highly diverse [17]. R.M. Wallen and coworkers investigated the gene–gene interaction between the *b* mating type genes and *ump2* encoding an ammonium transporter of *U. maydis*. They found that *bE* and *bW* were expressed to different levels in different haploid strains starved of ammonium, and that *bE* and *bW* had different effects on the expression of genes needed for mating and pathogenicity [18]. Finally, T. Kijpornyongpan and M.C. Aime compared physiology, reproductive biology, genomics, and molecular genetic knowledge of smuts and other dimorphic fungi to identify common cues and common signaling pathways, as well as commonalities in the role of the MAT locus in dimorphic switching and pathogenesis. This review revealed lipids and hydrophobicity as a potential common cue for dimorphic switching of plant-associated dimorphic fungi [19].

## 5. Conclusions

In summary, this issue shows that smut fungi harbor unexpected potential for the advancement of both science and technology. Exciting new discoveries have been made in the recent past, and with the increased attention are also expected for the future. We feel that this issue excellently highlights the great power of smut fungal research in the topics of biotechnology, biotrophy, and basic biology. Additionally, if after reading this issue you are still hungry for more, look out for the next Special Issue “Smut Fungi 2.0”, whose call for contributions is already open.
